# IgA nephropathy in a patient receiving infliximab for generalized pustular psoriasis

**DOI:** 10.1186/s12882-020-02015-0

**Published:** 2020-08-26

**Authors:** Yuka Segawa, Ryo Ishida, Fuminao Kanehisa, Kunihiro Nakai, Mari Morimoto, Masafumi Seno, Mayuka Nakayama, Tetsuro Kusaba, Norito Katoh, Keiichi Tamagaki

**Affiliations:** 1 Division of Nephrology, Department of Medicine, Kyoto Prefectural University of Medicine, 465 Kajii-cho, Kamigyo-ku, Kyoto, 602-8566 Japan; 2 Department of Dermatology, Kyoto Prefectural University of Medicine, 465 Kajii-cho, Kamigyo-ku, Kyoto, 602-8566 Japan

**Keywords:** IgA nephropathy, Generalized pustular psoriasis, Infliximab, TNFα, Secukinumab, IL-17

## Abstract

**Background:**

IgA nephropathy is the most common glomerulonephritis. Secondary IgA nephropathy complicated with systemic diseases, including psoriasis, is also often reported. Generalized pustular psoriasis is a form of psoriasis characterized by sterile pustules on reddened skin and fever. Infliximab, one of the first-line therapies for severe psoriasis, has also been reported to cause systemic vasculitis and IgA nephropathy. We herein report a case of IgA nephropathy activated during infliximab treatment for generalized pustular psoriasis.

**Case presentation:**

A 28-year-old woman presented with episodic gross hematuria, increasing proteinuria, and renal dysfunction. She had been receiving anti-TNFα therapy with infliximab because of generalized pustular psoriasis for 3 years, but her skin symptoms worsened following withdrawal during pregnancy. After delivery, her skin symptoms improved with the resumption of infliximab, but clinical signs suggested glomerulonephritis, and renal biopsy showed active IgA nephropathy. Infliximab was discontinued, and the combination of corticosteroids, tonsillectomy, and secukinumab, an IL-17A inhibitor, improved both the skin symptoms and the glomerulonephritis.

**Conclusions:**

In our case, the activity of IgA nephropathy was exacerbated by anti-TNFα therapy but was improved by the combination of corticosteroids, tonsillectomy, and an IL-17A inhibitor against the original disease. Autoimmune diseases may underlie the development of secondary IgA nephropathy associated with anti-TNFα therapy, and so further studies are needed to better understand the association between molecular-targeted drugs and IgA nephropathy.

## Background

IgA nephropathy (IgAN) is the most common glomerulonephritis. It manifests a variety of clinical courses, often shows persistent hematuria, and sometimes shows macroscopic hematuria associated with mucosal infections. IgAN is an autoimmune disorder in which IgA1-IgG immune complexes deposit on the mesangium and cause inflammation. The immunogenicity of IgA1 is due to an IgA1 galactosylation defect [1]. Secondary IgAN complicated with systemic diseases, such as liver cirrhosis, rheumatoid arthritis, inflammatory bowel disease, and other autoimmune diseases, is also often reported [2].

Generalized pustular psoriasis (GPP) is a form of psoriasis characterized by the presence of sterile pustules on reddened skin covering almost the entire body, frequently accompanied by fever. The prevalence of GPP is only about 1% among cases of psoriasis. It has been emphasized that psoriasis is a systemic chronic inflammatory disease rather than a skin disease [3, 4].

We report a case of IgAN activated during infliximab treatment for GPP and discuss the relationships among the two diseases and anti-TNFα therapy.

## Case presentation

**Fig. 1:**
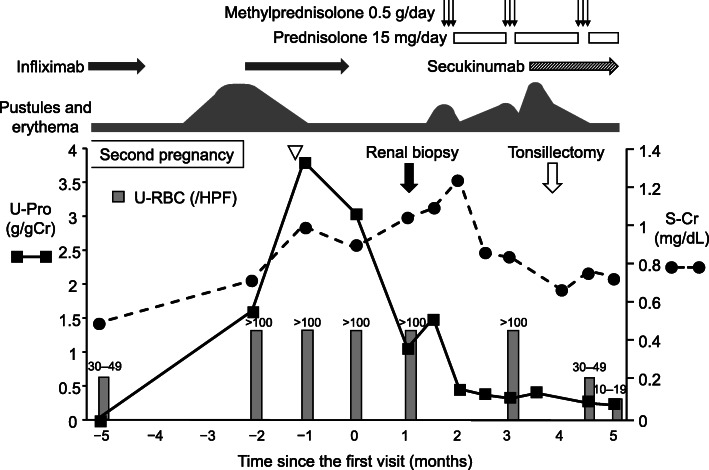
Clinical course. After second child birth, macroscopic hematuria, increase in urinary protein, and elevation of serum creatinine levels occurred with an upper respiratory infection (white arrowhead). S-Cr: serum creatinine, U-Pro: urinary protein, U-RBC: urinary red blood cells

A 28-year-old woman was referred to our hospital for episodic gross hematuria, increasing proteinuria, and renal dysfunction. She was diagnosed with GPP at the age of 2 years. Her father also suffered from GPP. Although GPP remained in remission for a long time, her skin symptoms deteriorated with pregnancy at the age of 24, around the same time as microscopic hematuria appeared. Corticosteroids were started, and both the skin symptoms and urinary findings improved after delivery. Anti-TNFα therapy with infliximab was initiated at the age of 25. Although her skin symptoms were relieved by the anti-TNFα therapy, she experienced two episodes of gross hematuria associated with throat infections. At the age of 27, her skin symptoms deteriorated when infliximab was discontinued during her second pregnancy, but improved with the resumption of infliximab after childbirth. Serum creatinine levels, hematuria, and urinary protein gradually worsened with upper respiratory infection (from 0.5 mg/dl, 30–49 /HPF, and negative, to 0.87 mg/dl, > 100 /HPF, and 3.04 g/gCr, respectively) (Fig. 1). When she first visited our hospital, there was mild enlargement of the bilateral palatine tonsils on physical examination. There were no findings suggesting IgA vasculitis such as fever, purpura, arthralgia, or abdominal pain. Her blood pressure was 107/65 mmHg. There were no medications other than infliximab. The laboratory workup performed on her first visit showed hematuria, proteinuria, and mild renal dysfunction (Table 1).

**Table 1:** Laboratory data at first visit

**Peripheral blood**		
WBC	6500	/μL
RBC	380 × 10^4^	/μL
Hb	11.0	g/dL
Hct	33.6	%
Plt	30.7 × 10^4^	/μL
**Blood chemistry**		
TP	6.5	g/dL
Alb	3.7	g/dL
AST	12	U/L
ALT	7	U/L
LDH	180	U/L
UA	4.9	mg/dL
BUN	25.5	mg/dL
Cr	0.87	mg/dL
Na	142	mEq/L
K	4.1	mEq/L
Cl	108	mEq/L
Ca	8.8	mg/dL
IP	4.2	mg/dL
CRP	0.03	mg/dL
HbA1c	5.8	%
**Serological tests**		
IgG	1077	mg/dL
IgA	332	mg/dL
IgM	128	mg/dL
**Serological tests**		
C3	87.8	mg/dL
C4	30.7	mg/dL
CH50	45.3	mg/dL
ANA	< 1:40	
MPO-ANCA	< 1.0	/mL
PR3-ANCA	2.0	/mL
**Urinalysis**		
Gravity	1.017	
pH	6.0	
Protein	3+	
Occult blood	3+	
**Sediments**		
RBC	> 100	/ HPF
WBC	1–4	/ HPF
Epithelial cells	1–4	/ HPF
**Urine chemistry**		
Protein	3.04	g/gCr
NAG	15.5	U/gCr
β2-m	216	μg/gCr
**Casts**		
Epithelial	5–9	/WF
RBC	1–4	/WF
Granular	1–4	/WF
Hyaline	1–4	/WF

**Fig. 2:**
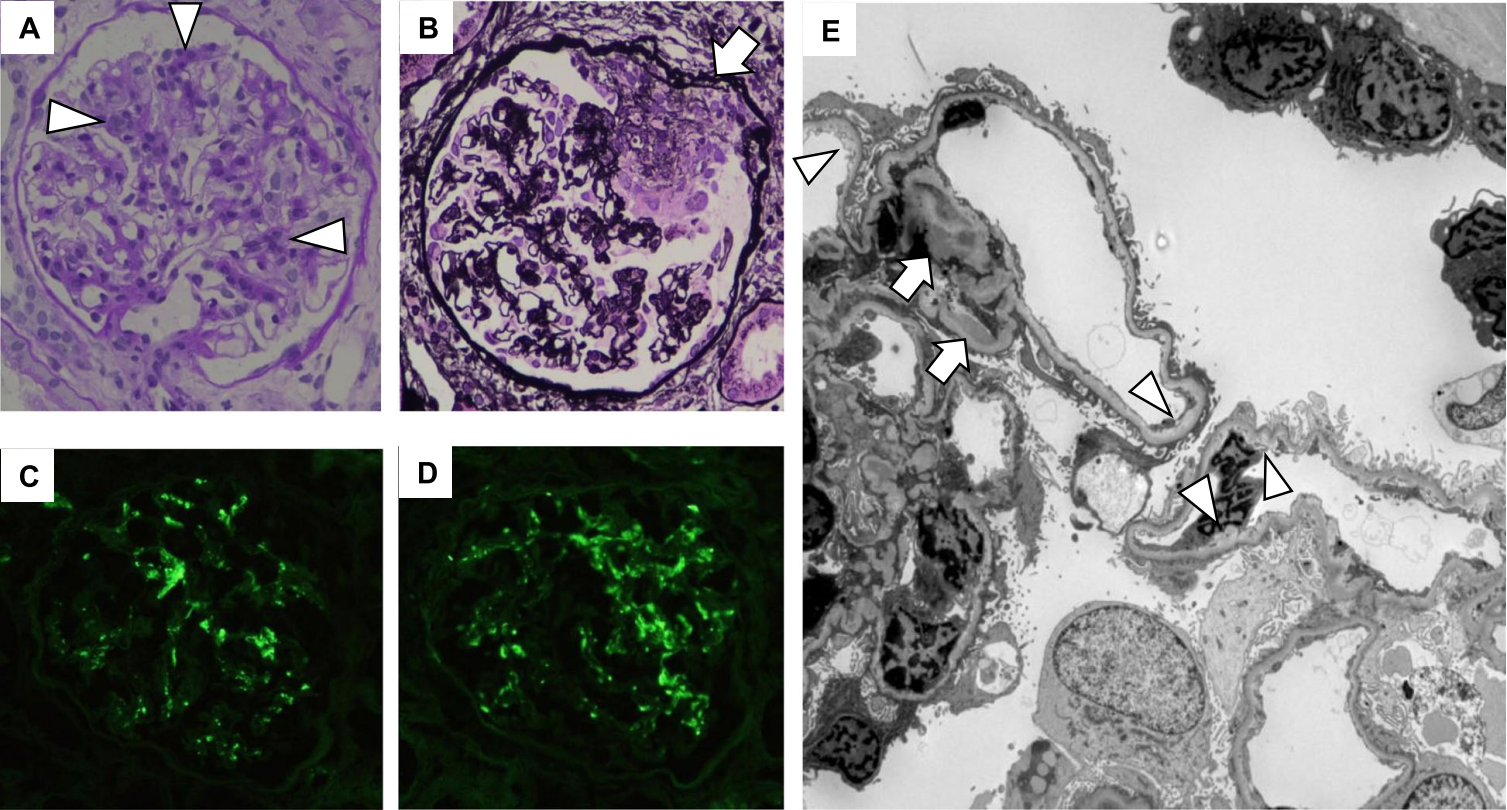
Findings of renal biopsy. Light microscopy showed segmental moderate mesangial hypercellularity (arrowheads in **a**: PAS stain × 400) and crescent formation including cellular crescent (arrow in **b**: PAM stain × 400). Immunofluorescence showed positive staining for IgA and C3 in mesangial areas (**c**: IgA, **d**: C3 × 400). Electron microscopy revealed electron-dense deposits in mesangial and paramesangial areas (arrows in **e**: × 1500), and segmental moderate subendothelial edema (arrowheads in **e**)

A renal biopsy was performed to clarify the diagnosis at 3 years after the initiation of anti-TNFα therapy which was 1 month after the second course of infliximab was discontinued and a little over 2 months after the end of the second pregnancy. A total of 33 glomeruli were included in the renal biopsy samples. Most glomeruli showed segmental mesangial proliferation (Fig. 2a). Three glomeruli showed global sclerosis and one glomerulus showed segmental sclerosis. Seven glomeruli showed crescents, including three cellular (Fig. 2b), three fibrocellular, and one fibrous crescent. Interstitial fibrosis was found in 10% of the renal cortex. There was slight arteriosclerosis but no vasculitis. Immunofluorescence microscopy showed positive staining for IgA and C3 in the mesangial areas (Fig. 2c, d). On electron microscopy, electron-dense deposits were evident in the mesangial and paramesangial areas, and segmental moderate subendothelial edema was observed (Fig. 2e).

The diagnosis was IgA nephropathy: M1 E0 S1 T0 C1, according to the Oxford classification [5]. In addition, segmental subendothelial edema on electron microscopy suggested the effect of pregnancy.

After discontinuing infliximab and observing whether serum creatinine and urinary findings improved, intravenous corticosteroid pulse therapy was started 28 days after the renal biopsy. Intravenous methylprednisolone for 3 days at a dose of 0.5 g daily was given for three courses every 40 days. As a follow-up treatment, prednisolone at a dose of 30 mg every other day was administered, but the dose was changed to 15 mg daily because of postprandial hyperglycemia and considering that she was lactating. Serum creatinine was reduced but the urine findings did not sufficiently improve. Inflammation of the skin was initially relieved by the first pulse therapy, but the oral corticosteroid was not sufficient to maintain her skin condition. Pustules and erythema worsened despite the second pulse therapy. Secukinumab, an anti-IL-17A monoclonal antibody, was initiated and administered at 300 mg subcutaneously at weeks 0, 1, 2, 3, and then every 4 weeks, which controlled the skin symptoms. Bilateral tonsillectomy was performed 93 days after the renal biopsy. Serum creatinine decreased to the normal range and urine findings gradually improved (Fig. 1). The patient was transferred due to relocation, and her urinary protein and urinary red blood cells remained at low levels.

## Discussion and conclusion

There are some reports that psoriasis is associated with glomerular disease. IgAN is the most frequent [6], and mesangial proliferative nephritis [7], amyloidosis [8], focal segmental glomerulosclerosis [9], minimal change disease [10], and membranoproliferative glomerulonephritis [11] have also been reported. However, as far as GPP is concerned, there are few reports of glomerulonephritis. Secondary IgAN often worsens or improves with the activity of the original disease [6, 12], but sometimes it does not [13, 14]. In the present case, the initial onset of hematuria and its response to glucocorticoids were consistent with the skin symptoms, and there was no macroscopic hematuria with upper respiratory infection. However, after starting anti-TNFα therapy, the clinical activity of IgAN continued to deteriorate without relation to the skin disease, and there was macroscopic hematuria with upper respiratory infection. This clinical course suggested that anti-TNFα therapy exacerbated IgAN and that IgAN was associated with GPP. In addition, pregnancy is obviously considered to be an exacerbatory factor in GPP, and could be related with proteinuria through endothelial dysfunction. Therefore, pregnancy may have contributed to the increased activity of GPP and possibly IgAN in our case.

The guidelines of the Medical Board of the National Psoriasis Foundation recommend acitretin, cyclosporine, methotrexate, or infliximab as first-line therapies for adult GPP. Although the data are limited, first-line therapy for pregnant women with GPP includes cyclosporine, oral corticosteroids, and topical agents. Because of its rapid onset of effect, infliximab is considered the first-line treatment especially in patients with severe and extensive disease [15]. Although tonsillectomy is not a standard treatment for GPP, there are some reports of its efficacy in psoriasis [16–18]. Therefore, tonsillectomy was performed as a possible treatment for both IgAN and GPP. In recent years, successful treatments of GPP with various biologic agents have been reported. Most recently, secukinumab, an IL-17A inhibitor, has shown promising ability to resolve symptoms rapidly in the treatment of GPP [3]. The efficacy of secukinumab has been shown in an interventional study [4], and its use is expected to increase.

Infliximab is an anti-TNFα monoclonal antibody that binds TNF with high affinity and has been widely used in recent years as a therapeutic agent for autoimmune diseases, with few side effects. As for pustular psoriasis, the TNFα inhibitors are central therapeutic agents as mentioned above [3, 15]. However, TNFα inhibitors have been reported to induce systemic vasculitis [19] and several types of glomerulonephritis, such as minimal change disease, membranous nephropathy, IgA nephropathy, pauci-immune necrotizing GN, and lupus-like nephritis [13, 20–24]. The mechanisms of vasculitis associated with anti-TNFα therapy are unclear, although the following hypotheses have been suggested: 1) TNFα / anti-TNFα immune complexes may deposit on small vessels and induce local complementary activation; and 2) the cytokine imbalance, with a shift from a Th1 to a Th2 profile, may promote the development of manifestations related to antibody-mediated immunity [25]. For glomerulonephritis, it is hypothesized that immune complexes are formed by a cross-reaction of the galactose-deficient IgA1 (Gd-IgA1) and the anti-drug antibodies against the glycan structures of TNFα inhibitors. These deposit on the mesangium, leading to IgAN [26]. In addition, the reported rate of detection of antinuclear antibodies in patients treated with anti-TNFα therapy is 29–76.7%. The immunological abnormalities can induce glomerulonephritis, such as membranous glomerulonephritis and lupus nephritis [23, 27]. In our case, macroscopic hematuria with upper respiratory infection and proteinuria became obvious after the initiation of anti-TNFα therapy, suggesting that underlying IgAN may have been exacerbated due to acceleration of IgA1 deposition by the TNFα inhibitor.

IL-17 expression increases locally in the skin in pustular psoriasis [28], and its inhibition is indicated as a specific treatment. IL-17 and keratinocytes are key factors in the development of psoriasis [29]. IL-17-producing CD4+ helper T (Th17) cells create a self-amplifying response that is markedly augmented in the presence of TNF-α, and Th17 cells produce high amounts of TNF-α [30–32]. Therefore, IL-17 and TNF-α are major drivers in the pathogenesis of psoriasis. In IgA nephropathy, IL-17 is also a key mediator of inflammation. IL-17 is considered to induce Gd-IgA1 production leading to the development of IgAN [33]. In addition, an imbalance of the regulatory T cell to Th17 cell ratio in IgA nephropathy patients has been suggested to play a role in disease pathogenesis and progression [34]. Moreover, serum/urinary IL-17A is elevated in IgAN patients compared with other nephropathy and control groups [35]. Thus, the inhibition of IL-17A may be effective not only against psoriasis but also against IgAN to some extent. Indeed, the urinary findings that were not improved by corticosteroids alone improved after the initiation of secukinumab in our case, which suggests the efficacy of the IL-17A inhibitor.

In conclusion, we experienced a case of IgAN associated with GPP. The activity of IgAN was exacerbated by anti-TNFα therapy but was improved by the combination of corticosteroids, tonsillectomy, and an IL-17A inhibitor against the original disease. Autoimmune diseases may underlie the development of secondary IgAN associated with anti-TNFα therapy, and so further studies are needed to better understand the association between molecular-targeted drugs and IgAN.

## Data Availability

Not applicable.

## Abbreviations

IgAN: IgA nephropathy
GPP: Generalized pustular psoriasis
